# Early Orthodontic Treatment in Children: A Qualitative Exploration of Orthodontists’ Perspectives and Practices in Saudi Arabia

**DOI:** 10.3390/healthcare14172679

**Published:** 2026-08-23

**Authors:** Guna Shekhar Madiraju, Yousef Majed Almugla

**Affiliations:** 1 Faculty in Pediatric Dentistry, Department of Preventive Dental Sciences, College of Dentistry, King Faisal University, Al Ahsa 36362, Saudi Arabia; 2 Faculty in Orthodontics, Department of Preventive Dental Sciences, College of Dentistry, King Faisal University, Al Ahsa 36362, Saudi Arabia; yalmugla@kfu.edu.sa

**Keywords:** early orthodontic treatment, interceptive orthodontics, mixed dentition, pediatric orthodontics, orthodontic decision-making, qualitative research, Saudi Arabia

## Abstract

**Background and Objectives:** The optimal timing of orthodontic intervention in children remains debated, particularly during the mixed dentition phase. Despite potential benefits, variability in clinical decision-making persists. This study explored orthodontists’ perceptions, clinical practices, and clinical decision-making regarding the timing of early orthodontic intervention in Saudi Arabia. **Materials and Methods:** An interpretive qualitative study was conducted using semi-structured interviews with nineteen licensed orthodontists practicing in Saudi Arabia. Participants were purposively sampled to ensure diversity in clinical experiences and practice settings. Interviews were conducted in English and Arabic, using detailed contemporaneous field notes that were subsequently expanded for analysis. Data were analyzed using thematic analysis following Braun and Clarke’s framework, with iterative coding supported by investigator triangulation, reflexive memoing, and participant validation. Data collection continued until thematic saturation was achieved. **Results:** Four major themes were identified. Early intervention was widely perceived as beneficial, particularly for functional problems, Class III malocclusions, and crossbites. However, decision-making was highly individualized, with many clinicians adopting a selective approach and deferring treatment in mild cases. Variation in clinical reasoning was observed across individual participant accounts, reflecting clinical judgment and contextual influences. Key barriers included limited parental awareness, financial constraints, and delayed referrals. System-level factors, such as variability in insurance coverage, referral pathways, and the lack of structured early screening programs, were identified as important influences on access to timely care. **Conclusions:** Early orthodontic intervention should be applied selectively, based on clinical indications and patient-specific factors. The context-specific findings highlight the need for appropriately tailored guidance, improved referral systems, structured early screening, and consideration of clinical, practitioner-related, and healthcare system factors to support consistent, patient-centered orthodontic care.

## 1. Introduction

The timing of orthodontic intervention in children remains an ongoing challenge in clinical practice, particularly during the mixed dentition phase when growth modification may be possible. Early orthodontic treatment is commonly considered for the management of developing malocclusions and may help guide craniofacial growth while addressing functional, esthetic, and psychosocial concerns [1,2]. Indications for such intervention include dental crowding, crossbites, Class II and Class III malocclusions, and deleterious oral habits [3,4,5].

Despite the potential benefits of early orthodontic treatment, determining the appropriate timing and indications for intervention remains challenging. Variability in treatment decisions may also reflect differences in practitioner training, clinical experience, interpretation of available evidence, and healthcare system factors [4,6,7,8]. Recent evidence syntheses and contemporary clinical reviews continue to highlight uncertainty regarding the optimal timing of treatment for several types of malocclusion. They suggest that treatment decisions should be individualized according to growth pattern, malocclusion severity, functional impairment, and patient-related factors rather than chronological age alone [9,10,11,12,13]. Previous studies conducted in Saudi Arabia have reported differences in orthodontic treatment preferences among dental practitioners, with early intervention more commonly considered for Class III malocclusions, whereas Class II cases are often managed at a later stage of dental development [7,14]. Orthodontists tend to adopt a selective approach, prioritizing early treatment for cases with clear skeletal or functional indications while postponing intervention in cases where long-term benefits remain uncertain [7]. Such variation in clinical decision-making may influence treatment pathways, resource utilization, and patient outcomes, highlighting the need to better understand the factors that shape orthodontists’ choices regarding early intervention.

In addition to clinical considerations, early orthodontic treatment is influenced by broader psychosocial and healthcare-related factors. Parental awareness, esthetic expectations, financial considerations, and access to specialist care may affect the timing of orthodontic consultation and treatment initiation [15,16]. In Saudi Arabia, orthodontic care is provided through a mixed healthcare system comprising public governmental services and private sector clinics. Referral pathways often vary across clinical settings during the mixed dentition phase. Access to orthodontic care may also be influenced by regional differences, workforce distribution, waiting times, and variability in insurance coverage. These structural and contextual factors make Saudi Arabia a particularly relevant setting for examining how clinical, systemic, and cultural influences intersect in early orthodontic decision-making.

Despite the availability of quantitative and survey-based studies in Saudi Arabia [4,7], evidence exploring the qualitative dimensions of orthodontists’ decision-making remains limited. Previous Saudi studies have primarily quantified treatment preferences and identified variations in the timing of treatment initiation among practitioners [7,14], but the underlying reasoning and experiences that shape these clinical decisions remain insufficiently explored. Broader dental and orthodontic literature suggests that these decisions involve complex professional judgments that cannot be fully captured through quantitative approaches alone [15].

Qualitative exploration is therefore needed to understand how orthodontists integrate clinical evidence, professional experience, patient-related considerations, and healthcare system factors when making decisions about early orthodontic treatment. To our knowledge, no qualitative study has specifically examined these decision-making processes within the Saudi Arabian healthcare context. Accordingly, the present study aimed to explore orthodontists’ perceptions, clinical practices, and decision-making regarding early orthodontic treatment in children in Saudi Arabia, with particular emphasis on the clinical, professional, and contextual factors that shape these decisions.

## 2. Materials and Methods

### 2.1. Ethical Considerations

Ethical approval for the study was obtained from the relevant institutional research ethics and review board (Ref: KFU-REC-2025-NOV-ETHICS3730). Participation was voluntary, and all participants provided informed consent prior to the interviews. Confidentiality and anonymity were maintained throughout the research process. Participants’ identifying information was excluded from interview records, and all data were securely stored and used exclusively for research purposes. All interview records were assigned alphanumeric codes to further protect participant identity. This study was reported in accordance with the Consolidated Criteria for Reporting Qualitative Research (COREQ), a 32-item checklist designed to enhance transparency and completeness in the reporting of qualitative studies involving interviews and focus groups.

### 2.2. Study Design

This study employed an interpretive qualitative research design to explore practicing orthodontists’ perceptions and clinical decision-making processes regarding early orthodontic treatment in children in Saudi Arabia. A qualitative approach was selected as it enabled in-depth exploration of clinicians’ experiences, professional reasoning, and contextual influences shaping treatment decisions, allowing for the capture of nuanced insights not readily accessible through quantitative methods.

**Figure 1:**
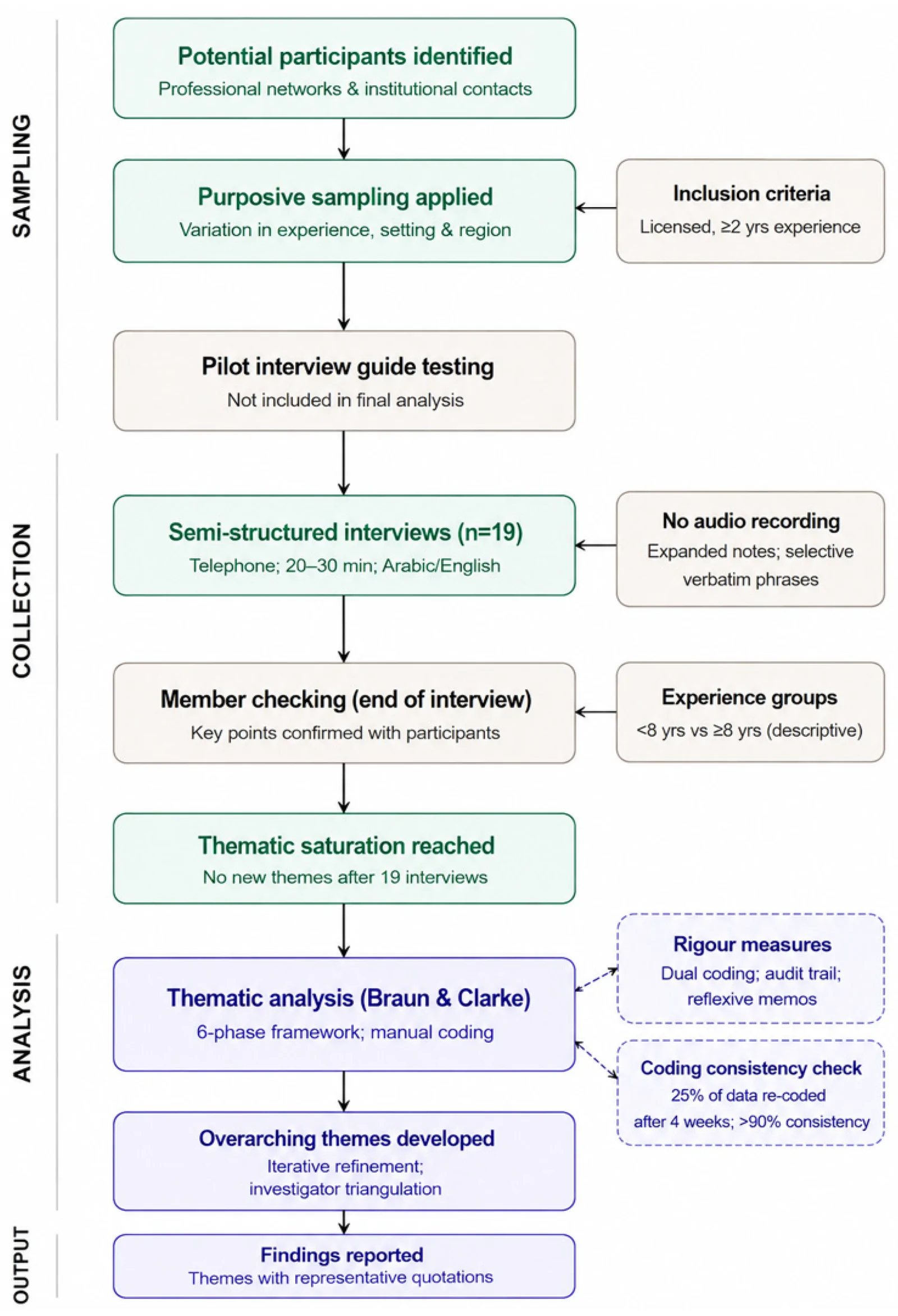
Flowchart of the study.

The study was guided by an interpretivist-constructivist paradigm, which recognizes that professional knowledge and clinical decision-making are shaped through individual experiences, interactions, and social contexts. From this perspective, reality is understood as multiple and context-dependent rather than singular and objective. Accordingly, the study explored how orthodontists interpret their clinical experiences and assign meaning to factors influencing early orthodontic treatment decisions. This paradigm emphasizes the importance of orthodontists’ perspectives, values, and professional experiences as valuable sources of knowledge for understanding complex clinical practices. The overall study workflow, including participant recruitment, data collection, and thematic analysis procedures, is illustrated in Figure 1.

Throughout the study, reflexive practices were maintained. The researchers remained aware of how their clinical backgrounds, professional experiences, and prior assumptions regarding early orthodontic treatment could influence data collection, interpretation, and analysis. Reflexive discussions and memoing were used throughout the analytical process to critically examine emerging interpretations and ensure that findings remained grounded in participants’ perspectives. Reflexivity is widely recommended in qualitative research to ensure that interpretations remain grounded in orthodontists’ perspectives rather than researchers’ assumptions [17].

### 2.3. Sampling and Recruitment

Orthodontists were recruited using purposive sampling to obtain diverse perspectives from clinicians with varying professional experiences and practice backgrounds. Efforts were made to include orthodontists with variation in years of clinical experience, workplace setting (public or private sector), and geographic regions across Saudi Arabia. These characteristics were considered to enhance the diversity of perspectives rather than for formal comparative analysis. Potential participants were identified based on predefined eligibility criteria and the desired variation in professional characteristics relevant to the study objectives.

The inclusion criteria were licensed orthodontists currently practicing in Saudi Arabia with at least two years of clinical experience. No additional exclusion criteria were applied other than unwillingness or inability to participate. It is acknowledged that recruitment through professional networks may have introduced selection bias, potentially favoring participants more engaged in academic or professional activities. Potential participants were approached through professional dental networks, institutional contacts, and professional communication platforms. Those who expressed interest received detailed information regarding the study objectives and procedures. Participation was voluntary, and participants were informed that their responses would remain confidential. Rapport was established before each interview to facilitate open and honest communication. Written informed consent was obtained from all participants prior to the interviews. A total of 25 orthodontists who fulfilled the eligibility criteria were identified and invited to participate. Of those invited, four declined participation and two did not respond, resulting in a final purposive sample of 19 orthodontists who completed the interviews.

For descriptive purposes, participants’ clinical experience was dichotomized into less experienced (<8 years) and more experienced (≥8 years) groups. This categorization was used to facilitate interpretation of variations in clinical perspectives observed during thematic analysis and to provide a balanced distribution of orthodontists across experience levels. The grouping was intended solely for descriptive contextualization and not for comparative evaluation.

### 2.4. Data Collection

Data were collected through semi-structured interviews using an interview guide developed based on the study objectives and previous literature regarding early orthodontic treatment and clinical decision-making [Supplementary File S1]. The interview guide was structured around key domains identified in the previous literature, including indications for early treatment, perceived effectiveness, treatment timing, parental acceptance, barriers to care, professional knowledge and clinical experience, and healthcare system influences. These domains were informed by previous research examining early orthodontic treatment, treatment timing, orthodontists’ clinical perspectives, parental factors, and factors influencing early orthodontic care [2,4,5,16]. This approach facilitated the generation of detailed accounts and supported a comprehensive understanding of orthodontists’ perspectives regarding indications for early treatment, treatment timing, clinical reasoning, perceived benefits and limitations, and contextual factors influencing decision-making. Prior to the main study, the interview guide was pilot-tested among a small group of orthodontists to assess the clarity, relevance, and sequencing of questions. Data from the pilot interviews were not included in the final analysis.

All interviews were conducted by the researcher (YA), a Saudi male orthodontist with academic and clinical experience in orthodontics and qualitative dental research. YA was fluent in both Arabic and English, allowing interviews to be conducted in the participant’s preferred language. The interviewer had no prior professional or personal relationship with the participants before the study. Familiarity with the local cultural and professional context facilitated communication and rapport with participants. Detailed contemporaneous notes were recorded (YA) during each interview, while another researcher (GS) subsequently organized and expanded the interview records with contextual details and reflective memos to ensure consistency and completeness of documentation across participants. These expanded records were used as the primary qualitative data source for analysis and did not constitute verbatim interview transcripts.

Semi-structured interviews were conducted via secure telephone calls at a mutually convenient time. Each participant completed a single semi-structured interview lasting approximately 20–30 min in Arabic or English according to participant preference, following a flexible interview guide that allowed additional probing where appropriate.

Before each interview, participants were informed about the option of audio recording and written consent for recording was requested. Although five participants agreed to audio recording, most declined because of privacy or confidentiality concerns and cultural considerations within the local context. To ensure methodological consistency in the primary data source across all interviews, all interviews were documented using detailed contemporaneous field notes rather than a combination of audio transcripts and field notes. The interviewer sought clarification of participants’ responses whenever required and summarized the key points at the end of each interview to allow participants to confirm that their views had been accurately captured. Immediately after each interview, the field notes were expanded with contextual details and reflective memos while the discussion remained fresh in memory. Interviews conducted in Arabic were translated into English for analysis. All translations were reviewed by a bilingual researcher to preserve the intended meaning and context of participants’ responses.

Participant recruitment continued until thematic saturation was achieved, defined as the point at which successive interviews yielded no substantially new codes, concepts, or themes relevant to the research objectives. Saturation was assessed iteratively by both researchers during data collection and manual thematic analysis through ongoing review of the developing codes and themes. Although a formal saturation grid was not maintained, the analytical process was documented through analytical memos and regular discussions between the researchers. Recruitment was discontinued after the nineteenth interview, when no substantially new codes or themes emerged, and all 19 interviews were included in the final analysis.

### 2.5. Data Analysis

Data were analyzed manually using thematic analysis guided by the six-phase framework of Braun and Clarke [18]. The interview records and expanded field notes were repeatedly reviewed to achieve familiarity with the data. Meaningful segments were systematically coded to identify key concepts related to orthodontists’ perceptions, clinical reasoning, and contextual influences on early orthodontic treatment decisions. Initial coding and development of preliminary themes were conducted manually by one researcher (GS) and the emerging codes and themes were independently reviewed by a second researcher (YA) to enhance credibility and consistency. Any disagreements in coding or theme interpretation were resolved through discussion and consensus. Data collection and analysis were conducted concurrently, allowing emerging insights to inform subsequent interviews and support iterative refinement of developing themes. Themes were subsequently reviewed, refined, and named to capture their central organizing concepts before representative excerpts were selected to illustrate each theme.

Trustworthiness was enhanced through investigator triangulation and reflexive memoing. Field notes and reflective memos were independently reviewed by both researchers during coding and theme development. Participant validation during the interviews, through clarification of responses and confirmation of key points at the conclusion of each interview, helped ensure that the documented data accurately reflected participants’ intended meanings. Coding discrepancies were resolved through discussion until consensus was achieved. A subset of interview records (25%) was re-coded after a four-week interval to assess coding consistency, demonstrating high intracoder consistency (>90%). Because the majority of interviews were not audio recorded, the illustrative excerpts presented in this manuscript were derived from contemporaneously documented and expanded field notes rather than verbatim interview transcripts.

## 3. Results

A total of 19 orthodontists participated in the study. Participant characteristics are summarized in Table 1. All participants were male, reflecting the composition of the recruited sample and the availability of eligible participants during the recruitment period. The participants represented diverse nationalities, with ages ranging from 29 to 47 years.

Participant responses were anonymized and assigned sequential identifiers (P1–P19). Analysis of the interview data generated four major themes reflecting orthodontists’ perceptions, clinical reasoning, and contextual influences related to early orthodontic intervention. These themes are presented below, with illustrative excerpts from the documented and expanded interview field notes. Short verbatim expressions are included only when exact wording was documented; other excerpts were reconstructed or paraphrased to represent participants’ views and are not presented as verbatim quotations.

### 3.1. Theme 1: Perceptions of Early Orthodontic Benefits

Most orthodontists generally considered early orthodontic intervention as beneficial when applied in appropriately selected cases, particularly for managing functional problems and skeletal discrepancies during the growth period. They described early treatment as an opportunity to guide craniofacial development, improve functional outcomes, and potentially reduce the complexity of future orthodontic management.

Several participants highlighted the importance of addressing specific problems during the growth period, particularly conditions where early correction may influence functional adaptation or skeletal development.

*Early treatment was perceived as an opportunity to ‘guide jaw growth’ and potentially reduce the need for more complicated treatment later.*

(P3)

Participants frequently identified crossbites and growth-related discrepancies as conditions where early intervention could provide meaningful benefits.

*Early correction of crossbites was perceived to improve bite and function, support dental and skeletal development, and potentially ‘reduce the severity of orthodontic problems later’.*

(P7)

However, some clinicians noted that early treatment does not necessarily eliminate the need for additional orthodontic care during adolescence. They emphasized that a second phase of treatment may remain necessary, depending on growth changes and the development of the permanent dentition, to achieve optimal results.

*Early intervention was considered beneficial but not necessarily definitive, as some children may still require a second phase of treatment during adolescence.*

(P10)

### 3.2. Theme 2: Clinical Decision-Making and Case Selection

Participants consistently described early orthodontic treatment decisions as individualized and dependent on specific clinical indications rather than as a routine approach for all children. Clinical presentation, growth potential, functional concerns, and anticipated treatment outcomes were described as important considerations influencing treatment timing.

Functional crossbites and skeletal discrepancies were commonly identified as conditions for which early intervention was considered beneficial. Class III malocclusions and anterior crossbites were frequently cited as conditions where timely treatment during the growth phase could positively influence jaw development and improve outcomes.

*Early treatment was considered particularly appropriate for selected Class III cases, where intervention during growth may influence jaw development and improve treatment outcomes.*

(P3)

*Posterior and anterior crossbites were viewed as conditions that may respond favorably to intervention during the mixed dentition, with potential functional and skeletal benefits.*

(P12)

In contrast, several participants described adopting a more observational approach, preferring to monitor growth and delay treatment until the permanent dentition stage when clinically appropriate, particularly in cases of mild or borderline malocclusions (P11).

### 3.3. Theme 3: Barriers to Early Orthodontic Care

Participants identified several factors that may delay access to early orthodontic assessment and treatment. Limited parental awareness regarding the importance of early orthodontic evaluation was frequently described as a contributing factor to delayed presentation.

*Limited parental awareness was perceived as contributing to delayed orthodontic presentation, with some parents failing to recognize that orthodontic problems can be addressed “early”.*

(P3)

Financial considerations were also described as an important barrier, particularly for treatments requiring multiple phases or when insurance coverage was limited. Participants noted that financial concerns could influence parental decisions regarding treatment initiation and continuation.

*Treatment costs and limited insurance coverage were described as a major consideration for families, particularly when early intervention involved multiple phases of treatment.*

(P5)

Participants also highlighted delayed referrals from general dental practitioners as a factor affecting timely orthodontic assessment. Some participants perceived that inconsistent recognition of developing malocclusions may contribute to postponed referrals.

*Delayed referral from general dental practitioners was perceived as another factor contributing to delays in timely orthodontic assessment and intervention, particularly when “early signs of malocclusion” were not recognized.*

(P12)

### 3.4. Theme 4: Contextual Influences on Early Orthodontic Practice

Participants described early orthodontic decision-making as influenced not only by clinical factors but also by professional experiences and broader healthcare contexts. Variation in perspectives was observed across individual participant accounts. In one account, a more cautious approach to early treatment was described, particularly for mild problems, while early intervention remained favored for selected Class III cases (P9). These observations were descriptive and were not intended to represent a formal comparison according to clinical experience.

Participants also discussed the possible influence of professional training backgrounds and clinical environments on treatment perspectives. Differences in exposure to early orthodontic concepts and growth modification approaches were mentioned by some participants, although these factors were not formally analyzed in the present study. Therefore, observations regarding training-related patterns should be interpreted cautiously, and may inform future research.

Beyond individual practitioner factors, participants highlighted healthcare system influences affecting access to early orthodontic care. Limited insurance coverage, variability in referral pathways, and absence of structured screening programs were described as barriers to timely assessment. Some participants also suggested that improved referral systems, supportive healthcare policies, and greater access to orthodontic services could facilitate earlier identification and management of children requiring intervention.

*School-based screening was suggested as a means of ‘picking up orthodontic problems earlier’ and facilitating timely referral of affected children.*

(P3)

*National guidelines and improved insurance coverage were also perceived as potential mechanisms for improving access to early orthodontic care and reducing barriers for families.*

(P5)

## 4. Discussion

This qualitative study explored orthodontists’ perceptions, clinical reasoning, and contextual considerations regarding early orthodontic intervention in children in Saudi Arabia. The findings provide insight into how orthodontists interpret evidence, evaluate clinical indications, and integrate patient-related and healthcare-related factors when deciding whether to initiate early treatment. Although participants generally recognized the potential benefits of early intervention for selected conditions, they consistently emphasized that treatment decisions should be individualized rather than routinely applied to all children.

Previous research examining early orthodontic treatment in Saudi Arabia has largely focused on treatment preferences, awareness, and parental perspectives [16,19]. The present study extends this evidence by exploring the reasoning processes underlying orthodontists’ decisions and highlighting the interaction between clinical indications, practitioner perspectives, and healthcare system factors. The findings suggest that early orthodontic intervention is viewed as a selective clinical strategy influenced by both evidence-based considerations and contextual realities within the Saudi healthcare environment.

### 4.1. Perceptions of Early Orthodontic Benefits

Participants generally perceived early orthodontic intervention as beneficial when applied to appropriately selected cases, particularly those involving functional disturbances, crossbites, and skeletal discrepancies. These perceptions align with established orthodontic principles, which suggest that early treatment may provide advantages in specific conditions where growth modification, correction of functional shifts, or improvement of occlusal relationships can be achieved. This finding is consistent with previous studies conducted in Saudi Arabia [4,7], and in other regions, where early intervention is frequently recommended for conditions such as Class II malocclusion and crossbite [5].

However, participants also acknowledged that early treatment does not necessarily eliminate the need for comprehensive orthodontic treatment during adolescence. This perspective reflects the existing evidence demonstrating that although early intervention may provide short-term improvements, the long-term advantages over later treatment remain condition-dependent and are not consistently demonstrated across malocclusion types. For example, studies evaluating early versus later treatment approaches have reported limited long-term differences for several conditions, despite improvements in intermediate outcomes such as overjet reduction or correction of specific functional problems [9,20,21,22]. Participants’ recognition that early treatment may represent an initial phase rather than a complete treatment solution highlights their awareness of the balance between potential benefits and realistic treatment expectations.

The findings also suggest that clinicians consider early intervention valuable when treatment timing coincides with biological opportunities, particularly growth-related changes. However, the perceived benefits were not interpreted as universal advantages, reinforcing the importance of appropriate case selection.

### 4.2. Clinical Decision-Making and Case Selection

A key finding of this study was the emphasis placed by participants on individualized treatment planning. Orthodontists consistently reported that early orthodontic intervention should be determined on a case-by-case basis, guided by clinical presentation, growth potential, functional needs, and anticipated treatment outcomes rather than a routine approach based solely on chronological age [23]. This reflects the complex nature of orthodontic decision-making, where clinicians must integrate available evidence with patient-specific characteristics and clinical judgment.

Participants frequently identified Class III malocclusions and anterior or posterior crossbites as conditions where early treatment may provide meaningful benefits. These findings are consistent with previous literature suggesting that early management may be particularly relevant for developing skeletal discrepancies and functional problems, where timely intervention during growth may improve treatment possibilities and reduce the severity of developing malocclusions [5,23]. Conversely, participants described a more cautious approach for mild or borderline malocclusions, often favoring observation and periodic reassessment before initiating active treatment. This selective approach aligns with previous studies from Saudi Arabia and other regions, which have reported variability in treatment timing based on case severity, practitioner judgment, and interpretation of clinical indications [4,7,24,25,26].

The findings also highlight the ongoing uncertainty regarding the optimal timing of orthodontic intervention. Routine early treatment is not strongly supported in the absence of clear clinical indications, particularly before the age of 10 years [13]. Meta-analytic evidence further supports this selective approach, indicating no consistent long-term advantage of early over later treatment [9]. Participants’ emphasis on selective intervention and careful case assessment therefore aligns with current evidence supporting an indication-based approach rather than universal early treatment. These findings highlight the need for clearer guidance regarding borderline cases during the mixed dentition phase. Context-specific clinical recommendations may assist orthodontists in integrating evidence, clinical expertise, and patient-related factors while maintaining flexibility for individual treatment needs.

The interpretation of these findings should also consider the characteristics of the study sample. All participants in the present study were male, and therefore the perspectives captured may not fully represent the experiences and clinical reasoning approaches of female orthodontists. Previous research in healthcare settings has suggested that clinician characteristics, including gender, may influence aspects of professional communication, information-sharing behaviors, and clinical interactions [27]. Although the present study was not designed to examine gender-related differences in orthodontic decision-making, future research incorporating more gender-diverse samples may provide further insight into whether clinician characteristics influence approaches to early orthodontic treatment.

### 4.3. Barriers to Early Orthodontic Care

Participants identified several barriers that may influence timely access to early orthodontic assessment and intervention, including limited parental awareness, financial constraints, delayed referrals, and healthcare system-related challenges. These findings highlight that decisions regarding early orthodontic treatment are influenced not only by clinical indications but also by factors affecting whether children are assessed and treated at an appropriate developmental stage. This is consistent with previous studies conducted in Saudi Arabia, which have shown that access to orthodontic care is influenced by socioeconomic factors, referral pathways, and broader healthcare system dynamics [4,7,28].

Limited parental awareness regarding the importance of early orthodontic evaluation was frequently described as a contributor to delayed presentation. Previous studies have similarly demonstrated that parental knowledge and perceptions influence orthodontic consultation patterns and treatment timing [16]. Delayed referrals from general dental practitioners were also identified as a concern, reflecting variability in recognition of developing malocclusions and referral practices. Similar variations in orthodontic treatment timing and referral patterns have been reported in Saudi Arabia [4,7].

Financial considerations were another important barrier affecting access to early orthodontic care, particularly for treatments involving multiple phases or when insurance coverage is limited. Within Saudi Arabia’s mixed healthcare system, variations in insurance coverage, waiting times, referral pathways, and access to specialist services may further affect timely orthodontic assessment. Cultural influences, including family-centered decision-making and parental esthetic expectations, may also shape perceptions regarding the urgency of seeking early orthodontic care. Previous studies have similarly shown that socioeconomic factors and healthcare accessibility influence orthodontic treatment utilization and care-seeking behaviors [16,29]. Collectively, these factors may increase treatment complexity and limit the potential benefits of early intervention.

Addressing these barriers requires a multifactorial approach involving improved parental education, enhanced communication between general dentists and orthodontists, clearer referral pathways, and healthcare policies supporting appropriate access to orthodontic assessment.

### 4.4. Contextual Influences on Early Orthodontic Practice

The contextual influences identified in this study encompass both professional and healthcare system factors that shape early orthodontic decision-making. Participants described early orthodontic decision-making as influenced by professional experiences, practice environments, and broader healthcare system factors. While some emphasized the potential benefits of intervening during growth, others favored a more cautious approach when clear clinical indications were absent, considering long-term stability and the possibility of relapse. This variation reflects the balance between proactive management and avoidance of unnecessary treatment and is consistent with previous research in Saudi Arabia reporting variability in orthodontic treatment approaches [7] and with the multifactorial nature of orthodontic decision-making [24].

Healthcare system factors, including insurance coverage, referral pathways, and the availability of structured screening programs, were also perceived to influence access to early orthodontic care. These findings suggest that improving early orthodontic care requires not only clinician-level decision-making but also broader system-level strategies [1]. Overall, these findings underscore the need to integrate clinical evidence with contextual considerations to support consistent yet adaptable approaches to early orthodontic intervention.

### 4.5. Strengths

This study offers in-depth qualitative insight into orthodontists’ perceptions and clinical decision-making regarding early orthodontic intervention in children. By exploring practicing clinicians’ perspectives, the study provides a more nuanced understanding of how treatment decisions are formed in routine clinical practice. A major strength lies in the inclusion of orthodontists practicing in Saudi Arabia with varied professional and educational backgrounds, including both Saudi and non-Saudi practitioners. This diversity enabled the exploration of a broad range of viewpoints shaped by differences in training, clinical experience, and practice context within the Saudi healthcare system. In addition, the study identified several clinical and system-level influences that shape early orthodontic decision-making. These findings contribute context-specific insights that may support efforts to improve access to and delivery of early orthodontic services.

Although several individual factors identified in this study have been reported previously, the present study extends existing evidence by providing an in-depth qualitative exploration of how orthodontists integrate multiple contextual influences when making decisions regarding early orthodontic intervention. Rather than examining isolated treatment preferences, the findings illustrate the dynamic interplay among clinical indications, practitioner experiences, patient-related considerations, and healthcare system influences. The resulting conceptual thematic framework provides a context-specific understanding of clinical decision-making that may inform future qualitative research, clinical guidance, and healthcare planning within the Saudi Arabian context.

### 4.6. Limitations

Several limitations should be considered when interpreting these findings. First, the study was conducted among orthodontists practicing in Saudi Arabia and involved a relatively small sample, although thematic saturation was achieved. Consequently, the findings should be interpreted with appropriate consideration of their transferability to other healthcare systems and cultural contexts. Second, all participants in the recruited sample were male, as female orthodontists were not included due to local cultural and recruitment constraints during the study period. Consequently, the perspectives of female orthodontists were not represented, which may have limited the diversity of viewpoints captured. Future research should include more gender-diverse samples to explore a broader range of clinical perspectives. Third, data were collected through telephone interviews, which may have limited the observation of non-verbal cues despite facilitating participation from geographically diverse clinicians.

Fourth, recruitment through professional networks may have introduced selection bias, potentially favoring clinicians who were more engaged in professional activities or had a particular interest in early orthodontic care. Response bias is also possible, as participants may have described idealized rather than routine clinical practices. Finally, variations in perspectives related to clinical experience, practice setting, and training background were observed within individual accounts but were not formally compared. Future qualitative and mixed-methods research involving larger and more diverse samples could further explore how these contextual factors may relate to clinical decision-making regarding early orthodontic treatment.

### 4.7. Implications for Practice and Research

The findings of this study support a selective, indication-based approach to early orthodontic intervention, where treatment decisions are guided by clinical need, growth considerations, and patient-specific factors. Early treatment should be prioritized for cases with clear functional or skeletal indications, such as anterior or posterior crossbites, Class III malocclusions, and oral habits that may adversely affect craniofacial growth and development. In contrast, mild or borderline malocclusions may be managed through regular monitoring during the mixed dentition phase, with intervention initiated when clinically indicated.

The development of context-specific clinical guidance may help promote greater consistency in treatment planning while allowing flexibility for individual patient needs. Such guidance should consider the characteristics of the Saudi healthcare system, including referral pathways and access to specialist care. Given the variation in clinical perspectives identified among participants, these guidelines may support evidence-informed and standardized approaches to patient management. At the healthcare system level, improving early identification of orthodontic problems may require stronger collaboration between general dental practitioners, orthodontists, and public health services. School-based screening initiatives, improved referral systems, and increased parental awareness may facilitate timely and appropriate intervention.

From a research perspective, future studies should evaluate the long-term clinical outcomes, patient-reported outcomes, and cost-effectiveness of early versus delayed orthodontic treatment within the Saudi population. Further quantitative and mixed-methods studies examining the influence of practitioner-related factors, including clinical experience, training background, practice setting, and gender, would further enhance understanding of variability in orthodontic decision-making.

**Figure 2:**
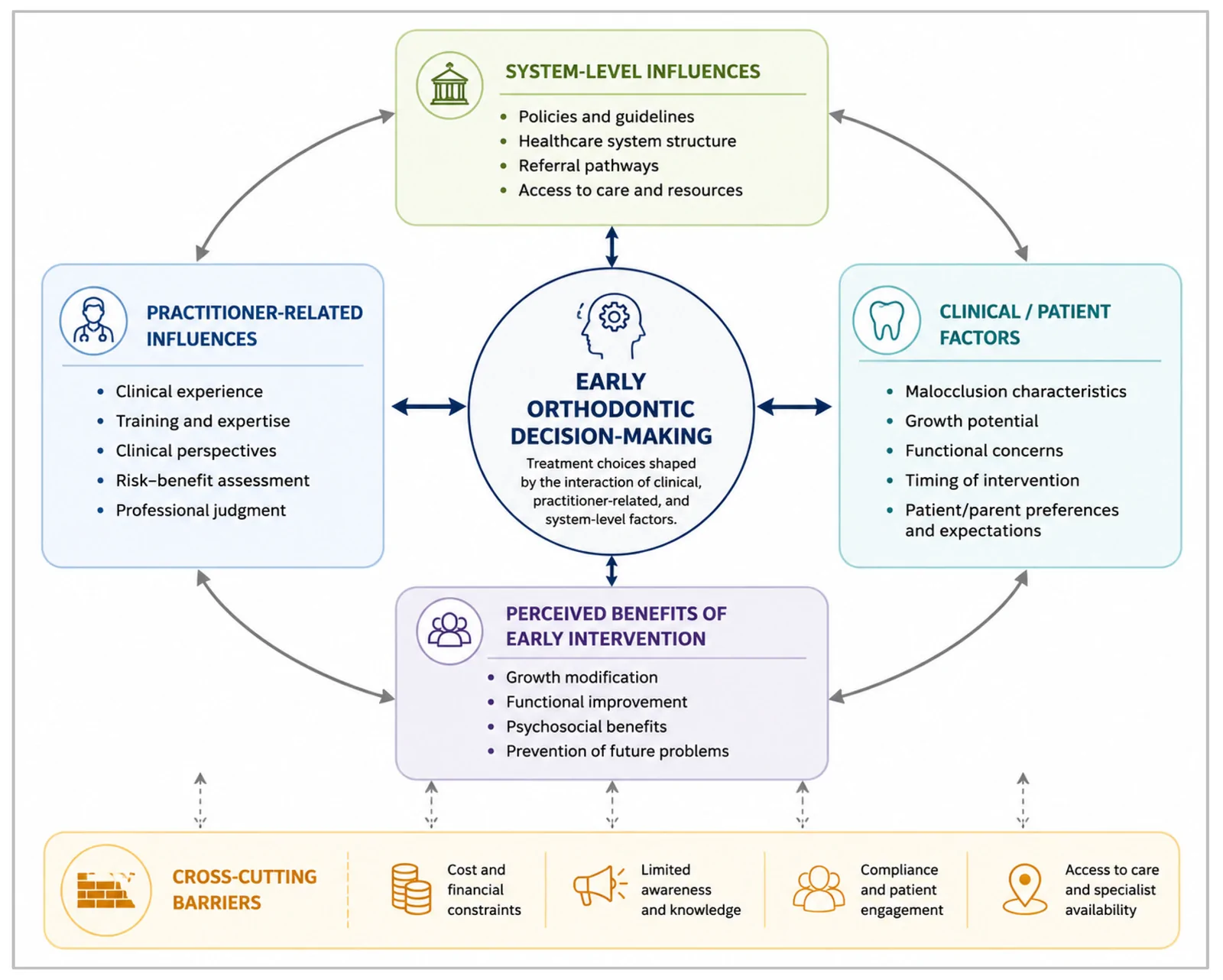
Emergent thematic model illustrating the key factors influencing early orthodontic decision-making in children.

To integrate the four themes identified through the thematic analysis, an interpretive conceptual model was developed (Figure 2). The model illustrates the dynamic interrelationships among clinical and patient factors, practitioner-related influences, perceived benefits of early intervention, healthcare system factors, and cross-cutting barriers that collectively shape early orthodontic decision-making. Rather than representing a causal framework, the model provides an interpretive synthesis of the study findings, highlighting the complexity of clinical decision-making and the interaction of multiple contextual influences. This conceptual framework may inform future research, healthcare planning, and the development of context-specific clinical guidelines for early orthodontic care.

## 5. Conclusions

This qualitative study provides insight into how orthodontists practicing in Saudi Arabia approach early orthodontic intervention in children. Early treatment was generally viewed as beneficial for selected cases with clear functional or skeletal indications; however, participants consistently emphasized an individualized, evidence-informed approach to treatment planning. Clinical decision-making was shaped by the interplay of clinical and patient factors, practitioner-related influences, and healthcare system factors, highlighting the complex and context-dependent nature of early orthodontic intervention. The findings highlight the need for context-specific clinical guidance, improved referral pathways, and strategies that support timely access to appropriate orthodontic care. Overall, this study contributes to a deeper understanding of orthodontic decision-making in the Saudi Arabian context and presents an interpretive conceptual framework that may inform future research, clinical practice, and healthcare policy.

## Figures and Tables

**Table 1:** General Characteristics of Participants (*n* = 19).

Characteristic	Category	Frequency *n* (%)
Gender	Male	19 (100)
Nationality	Saudi	11 (57.9)
	Non-Saudi	8 (42.1)
Clinical experience	<8 years	10 (52.6)
	≥8 years	9 (47.4)
Location of practice	Eastern region	4 (21.1)
	Central region	5 (26.3)
	Northern region	3 (15.8)
	Western region	4 (21.1)
	Southern region	3 (15.8)
Practice setting	Public sector	9 (47.4)
	Private sector	10 (52.6)

## Data Availability

The data presented in this study are available on request from the corresponding author. The data are not publicly available due to institutional restrictions.

## Supplementary Materials

The following supporting information can be downloaded at https://www.mdpi.com/article/10.3390/healthcare14172679/s1, Supplementary File S1: Annexure A (Interview Guide).
