# Supplementary material for: Early Orthodontic Treatment in Children: A Qualitative Exploration of Orthodontists’ Perspectives and Practices in Saudi Arabia
Source: Healthcare (Basel). 2026 Aug 23;14(17):2679. doi: 10.3390/healthcare14172679 (PMC13565339; doi:10.3390/healthcare14172679)
Supplement: Supplementary file 1 [file healthcare-14-02679-s001.zip › healthcare-4477434-supplementary.pdf]

Supplementary File S1

Annexure A

Perceptions and practices of Orthodontists in Saudi Arabia toward early orthodontic treatment in children

Dear Doctor,

You are invited to participate in a research study titled *"Perceptions and Practices of Orthodontists in Saudi Arabia Toward Early Orthodontic Treatment in Children."*

PARTICIPATION: Your participation in this study is entirely voluntary. You may decline to participate or withdraw from the interview at any time without any penalty. You are also free to refuse to answer any question you do not wish to answer, without providing a reason.

CONFIDENTIALITY: This study involves a structured interview conducted via telephone. No identifying information, such as your name, will be recorded during the interview. The telephone conversation will not be audio-recorded. Instead, the researcher will manually document your responses in written form. All collected information will be kept strictly confidential and used solely for research purposes. The data will later be reviewed, coded, and analyzed to identify common themes. At no stage will your identity be linked to your responses or disclosed in any reports or publications. All written notes will be stored securely and accessed only by the research team. The information collected will be analyzed in aggregate form to ensure anonymity.

CONTACT: f you have any questions or require further clarification about the study, please feel free to contact the principal investigator: [redacted] at Phone number # [redacted].

Consent Statement:
By clicking "I agree," you confirm that you have read and understood the information provided above. You voluntarily agree to participate in this study, including the telephone interview, and understand that you may decline to answer any question or withdraw from the study at any time without any consequences.

o I agree

Thank you in advance for your cooperation.

Interview Guide

"Thank you for agreeing to participate in this study".

Introduction:

"This interview aims to explore your views/perspectives and experiences regarding early orthodontic treatment in children, as well as any challenges or barriers you encounter when making decisions about providing such treatment.

We are speaking with a diverse group of licensed orthodontists practicing in Saudi Arabia, and your insights will help us better understand current clinical practices and identify areas for potential improvement in clinical guidelines and policy development. There are no right or wrong answers. We are interested in your personal experiences, opinions, and clinical decision-making processes. Please feel free to speak openly and honestly. If you have any questions at any point, I will be happy to clarify them. If you feel uncomfortable answering any question, you may choose to skip it, and we will move on to the next.

Please be assured that your responses will remain completely anonymous. No identifying information will be recorded, and your responses will be documented manually without audio recording. Any information you provide will be kept confidential and reported in a way that ensures that no individual participant can be identified. This interview will take approximately 20 to 30 minutes."

Warm-Up / Background Questions

- Can you briefly describe your current clinical role and the type of patients you usually treat?
- What types of malocclusions do you commonly encounter in children during the mixed dentition stage?

Perceptions of Early Orthodontic Treatment

1. What is your overall opinion regarding early orthodontic intervention in children?
2. Under what circumstances do you recommend early orthodontic treatment? If yes/No, then why or why not?
3. What types of cases/malocclusions/clinical conditions do you believe benefit most from early intervention?
4. How do you determine the appropriate timing for initiating early orthodontic treatment?
5. What clinical factors (e.g., growth potential, skeletal discrepancies, functional problems, patient cooperation) most influence your treatment decisions?
6. Can you describe an example of a case in which early orthodontic treatment was particularly successful or unsuccessful?
7. What do you consider to be the main limitations or advantages of early orthodontic treatment from your clinical perspective?
8. How do parents typically respond when you recommend early orthodontic treatment?
9. In your experience, what are the most common barriers to providing or accessing early orthodontic care?

   (Prompts: parental awareness, financial considerations, referral pathways, patient compliance, access to specialist care.)

Contextual Influences (Clinical Practice, Healthcare System, and Policy)

10. How do your clinical experience and professional training influence your decisions regarding early orthodontic treatment?

11. Do you think general dental practitioners receive sufficient training to recognize children who require early orthodontic referral?

12. Do you think routine orthodontic screening should be incorporated into school-based or primary healthcare programs in Saudi Arabia?

13. What changes could improve the delivery of early orthodontic care in Saudi Arabia?

14. Do you think national clinical guidelines, referral pathways, or insurance coverage could improve access to early orthodontic treatment?

Closing / Additional Insights:

15. Is there anything else you would like to add regarding early orthodontic treatment or clinical decision-making in Saudi Arabia?

الملحق أ

تصورات وممارسات أطباء تقويم الأسنان في المملكة العربية السعودية تجاه العلاج التقويمي المبكر للأطفال

عزيزي الطبيب،

ندعوكم للمشاركة في استبيان إلكتروني (رمز الاستجابة السريعة مرفق) حول بحثنا بعنوان "تصورات وممارسات أخصائيي تقويم الأسنان في المملكة العربية السعودية بشأن العلاج التقويمي المبكر للأطفال".

المشاركة: مشاركتكم في هذا الاستبيان اختيارية، ولكم الحق في رفض المشاركة أو الانسحاب في أي وقت دون أي عقوبة. كما يحق لكم رفض الإجابة عن أي سؤال لا ترغبون في الإجابة عنه لأي سبب كان.

السرية: تتضمن هذه الدراسة مقابلة منظمة تُجرى عبر الهاتف. لن يتم تسجيل أي معلومات تعريفية، مثل اسمكم، خلال المقابلة. ولن يتم تسجيل المكالمة الهاتفية صوتيًا. بدلاً من ذلك، سيقوم الباحث بتوثيق إجاباتكم كتابيًا. سيتم الحفاظ على سرية جميع المعلومات التي يتم جمعها واستخدامها لأغراض البحث فقط. سيتم لاحقًا مراجعة البيانات وتصنيفها وتحليلها لتحديد المواضيع المشتركة. لن يتم ربط هويتك بإجاباتك أو الكشف عنها في أي تقارير أو منشورات. سيتم حفظ جميع الملاحظات المكتوبة بشكل آمن ولن يتمكن من الوصول إليها سوى فريق البحث. سيتم تحليل المعلومات المجمعة بشكل إجمالي لضمان عدم الكشف عن هويتك.

للتواصل: إذا كانت لديك أي أسئلة بخصوص البحث، فلا تتردد في التواصل مع رئيس المجموعة، الدكتور [الاسم]، على الرقم [الرقم].

بالنقر على زر" أوافق"، فإنك تقر بما يلي :لقد قرأت وفهمت المعلومات المقدمة لك؛ وتوافق طواعية على أن تكون مشاركًا في هذه الدراسة، وتعلم أنه يمكنك رفض الإجابة على الأسئلة و/أو الانسحاب من البحث في أي وقت، دون الحاجة إلى تحديد الأسباب.

o أنا أوافق

نشكركم مقدمًا على تعاونكم.

الجزء ب: دليل المقابلة المتعمقة

مقدمة

شكرًا لمشاركتك. تهدف هذه المقابلة إلى استكشاف آرائك وتجاربك فيما يتعلق بالعلاج التقويمي المبكر للأطفال، بالإضافة إلى العوائق والعوامل التي تواجهها عند اتخاذ قرارك بشأن تقديم هذا العلاج. يُرجى المشاركة بصراحة؛ ستُحفظ هويتك سرًا.

نُجري مقابلات مع مجموعة متنوعة من أطباء تقويم الأسنان المرخصين الممارسين في المملكة العربية السعودية، وستساعدنا آراؤكم على فهم الممارسات العملية وتحديد مجالات التحسين المُحتملة في الإرشادات السريرية وتطوير السياسات. لا توجد إجابات صحيحة أو خاطئة، فنحن مهتمون فقط بتجاربكم وآرائكم وعمليات اتخاذ القرار. أشجعكم على الإجابة بأكبر قدر ممكن من الصراحة والشفافية. أنا أخصائي رعاية صحية، وسأكون سعيدًا بالإجابة على أي أسئلة لديكم. إذا شعرتم بأي إزعاج أو فضّلتم عدم الإجابة، فلا بأس، وسننتقل إلى السؤال التالي.

يرجى العلم أن أي شيء تشاركونه في هذه المقابلة سيُعامل بسرية تامة. عند نشر النتائج، لن يكون بالإمكان تحديد هوية أي مشارك. تستغرق كل جلسة ما بين ٢٠ و٣٠ دقيقة.

أسئلة الإحماء / الخلفية

- هل يمكنك أن تصف بإيجاز دورك السريري الحالي ونوع المرضى الذين تعالجهم عادةً؟
- ما هي أنواع سوء الإطباق التي تصادفها عادةً عند الأطفال خلال مرحلة الأسنان المختلطة؟

التصورات والمواقف

1. للأطفال؟ المبكر التقويمي التدخل في عام بشكل رأيك ما

2.ولماذا؟ المبكر؟ بالعلاج شخصيًا توصي هل

3.ما هي أنواع الحالات/سوء الإطباق/الحالات السريرية التي تعتقد أنها تستفيد أكثر من التدخل المبكر

4.كيف تحدد التوقيت المناسب لبدء العلاج التقويمي المبكر

5.ما هي العوامل السريرية (مثل إمكانات النمو، والاختلافات الهيكلية، والمشاكل الوظيفية، وتعاون المريض) التي تؤثر بشكل كبير على قراراتك العلاجية؟

6. هل يمكنك وصف مثال لحالة كان فيها العلاج التقويمي المبكر ناجحًا أو غير ناجح بشكل ملحوظ؟

7. ما هي أبرز مزايا وعيوب العلاج التقويمي المبكر من وجهة نظرك السريرية؟

8. كيف يتفاعل الآباء عادةً عند توصيتك بالعلاج التقويمي المبكر؟

9. بحسب خبرتك، ما هي أكثر العوائق شيوعاً أمام توفير أو الحصول على الرعاية التقويمية المبكرة؟

(.مواضيع للنقاش: وعي الوالدين، الاعتبارات المالية، مسارات الإحالة، التزام المريض، الوصول إلى الرعاية المتخصصة)

التأثيرات السياقية (الممارسة السريرية، نظام الرعاية الصحية، والسياسات)

10. كيف تؤثر خبرتك السريرية وتدريبك المهني على قراراتك المتعلقة بالعلاج التقويمي المبكر؟

11. هل تعتقد أن أطباء الأسنان العامين يتلقون تدريبًا كافيًا للتعرف على الأطفال الذين يحتاجون إلى إحالة مبكرة للعلاج التقويمي؟

12. هل تعتقد أنه ينبغي إدراج الفحص التقويمي الروتيني ضمن برامج الرعاية الصحية المدرسية أو الأولية في المملكة العربية السعودية؟

13. ما التغييرات التي يمكن أن تُحسّن تقديم الرعاية التقويمية المبكرة في المملكة العربية السعودية؟

14. هل تعتقد أن المبادئ التوجيهية السريرية الوطنية، أو مسارات الإحالة، أو التغطية التأمينية يمكن أن تُحسّن الوصول إلى العلاج التقويمي المبكر؟

:الخاتمة / ملاحظات إضافية

هل هناك أي شيء آخر تود إضافته بخصوص العلاج التقويمي المبكر أو اتخاذ القرارات السريرية في المملكة العربية السعودية؟.15
